# Thyroid Antagonists (Perchlorate, Thiocyanate, and Nitrate) and Childhood Growth in a Longitudinal Study of U.S. Girls

**DOI:** 10.1289/ehp.1409309

**Published:** 2015-07-07

**Authors:** Nancy A. Mervish, Ashley Pajak, Susan L. Teitelbaum, Susan M. Pinney, Gayle C. Windham, Lawrence H. Kushi, Frank M. Biro, Liza Valentin-Blasini, Benjamin C. Blount, Mary S. Wolff, for the Breast Cancer and Environment Research Project (BCERP)

**Affiliations:** 1 Department of Preventive Medicine, Icahn School of Medicine at Mount Sinai, New York, New York, USA; 2 University of Cincinnati College of Medicine, Department of Environmental Health, Cincinnati, Ohio, USA; 3 Environmental Health Investigations Branch, California Department of Public Health, Richmond, California, USA; 4 Division of Research, Kaiser Permanente, Oakland, California, USA; 5 Division of Adolescent Medicine, Cincinnati Children’s Hospital Medical Center, Cincinnati, Ohio, USA; 6 National Center for Environmental Health, Centers for Disease Control and Prevention, Atlanta, Georgia, USA

## Abstract

**Background:**

Perchlorate, thiocyanate, and nitrate are sodium/iodide symporter (NIS) inhibitors that block iodide uptake into the thyroid, thus affecting thyroid function. Thyroid dysfunction can adversely affect somatic growth and development in children. To our knowledge, no studies have examined effects of NIS inhibitors on body size measures.

**Objective:**

We investigated associations between NIS inhibitors and childhood growth in 940 girls from the Puberty Study of the Breast Cancer and Environment Research Program.

**Methods:**

Urine samples collected from girls 6–8 years of age at enrollment (2004–2007) from New York City, greater Cincinnati, Ohio, and the Bay Area in California were analyzed for NIS inhibitors and creatinine (C). The longitudinal association between NIS inhibitors and anthropometric measures [height, waist circumference, and body mass index (BMI)] during at least three visits was examined using mixed effects linear models, adjusted for race and site.

**Results:**

Compared with girls in the low-exposure group (3.6, 626, and 500 mg/gC, median perchlorate, thiocyanate, and nitrate, respectively) girls with the highest NIS inhibitor exposure (9.6, 2,343, and 955 mg/gC, median perchlorate, thiocyanate, and nitrate, respectively) had slower growth in waist circumference and BMI but not height. Significant differences in the predicted mean waist circumference and BMI between the low- and high-exposure groups were observed beginning at 11 years of age.

**Conclusions:**

Higher NIS inhibitor exposure biomarkers were associated with reductions in waist circumference and BMI. These findings underscore the need to assess exposure to NIS inhibitors with respect to their influence on childhood growth.

**Citation:**

Mervish NA, Pajak A, Teitelbaum SL, Pinney SM, Windham GC, Kushi LH, Biro FM, Valentin-Blasini L, Blount BC, Wolff MS, for the Breast Cancer and Environment Research Project (BCERP). 2016. Thyroid antagonists (perchlorate, thiocyanate, and nitrate) and childhood growth in a longitudinal study of U.S. girls. Environ Health Perspect 124:542–549; http://dx.doi.org/10.1289/ehp.1409309

## Introduction

Disruption of thyroid function is one of the strongest mechanisms linking environmental exposures with adverse health outcomes (Werner et al. 2005). Perchlorate, thiocyanate, and nitrate are sodium iodide symporter (NIS) inhibitors that block iodide uptake into the thyroid and thus can affect thyroid function. As known, iodine is necessary for the synthesis of thyroid hormones. Thyroid hormones are essential for normal growth; they promote and modulate the effects of growth hormone (GH) secretion (Burstein et al. 1979), and insulin growth factor (IGF)–1 mediates many of the effects of GH (Miell et al. 1993). These NIS inhibitors are ubiquitous in the environment, leading to widespread human exposure, mainly through ingested food and water (Lau et al. 2013; Murray et al. 2008). Perchlorate is a naturally occurring anion that is formed in the atmosphere and is synthesized primarily as ammonium perchlorate for producing solid propellant for rockets, missiles, fireworks, and other explosives. It is also found in some crop fertilizers formerly used in the United States (Mendiratta et al. 1996). Thiocyanate is found in foods such as milk and vegetables (Laurberg et al. 2002; Michajlovski and Langer 1958). It is also the main metabolite of cyanide exposure coming from tobacco smoke and certain foods such as cassava and almonds (Buratti et al. 1997). Nitrates can occur naturally in food, such as green leafy vegetables, or can be added as preservative (in meat and fish).

Ecologic, experimental, and observational studies have examined relationships of perchlorate exposure with thyroid hormones in adults, pregnant women, adolescents, and infants (Brechner et al. 2000; Chang et al. 2003; Crump et al. 2000; Greer et al. 2002; Li et al. 2000) with mixed results. Associations have been observed between perchlorate and decreased levels of thyroxine (T_4_) and increased thyroid-stimulating hormone (TSH) (Blount 2006; Steinmaus et al. 2007), with the strongest associations in women with low iodine and high thiocyanate (Steinmaus et al. 2013). Associations have also been reported for high nitrate exposure with increased thyroid volume and thyroid disorders (Aschebrook-Kilfoy et al. 2012; Tajtáková et al. 2006; van Maanen et al. 1994) and increased TSH levels (van Maanen et al. 1994). Perchlorate, thiocyanate, and nitrate exposures are cause for concern given their potential to decrease iodide concentration in the thyroid. Iodine status may influence growth through its effect on the thyroid (Zimmermann 2007). Data from cross-sectional studies on iodine intake and childhood growth are mixed; most studies in iodine deficient (ID) areas show retarded height and decreased weight and bone maturation compared with children in nonendemic areas (Azizi et al. 1995; Bautista et al. 1982; Thurlow et al. 2006). Effects of lower-level perchlorate exposure have not been well studied in children, including effects on growth. Whether thyroid disruption will occur when iodine is adequate, and whether these chemical exposures can cause changes in growth are important questions. *In vitro* studies of NIS inhibitors indicate that perchlorate, thiocyanate, and nitrate act additively to inhibit iodide uptake (Tonacchera et al. 2004), thus suggesting exposures should be considered together. We *a priori* hypothesized that the thyroid antagonists perchlorate, thiocyanate, and nitrate would have inverse associations with growth, height, weight, waist circumference, and body mass index (BMI).

We examined whether exposure to NIS inhibitors measured at one time point were associated with height, waist circumference, and BMI trajectories during childhood in an established cohort group of young girls with adequate iodine intake.

## Methods

This project was carried out as part of the Breast Cancer and the Environment Research Project (BCERP), a longitudinal study that is investigating early life risk factors for pubertal maturation that may be related to later breast cancer risk. Subjects include 1,239 females age 6–8 years at baseline enrollment (2004–2007) with no underlying endocrine medical condition affecting metabolism or growth. The study was conducted at three sites: *a*) Icahn School of Medicine at Mount Sinai Medical Center (NYC), which recruited only black or Hispanic girls in East Harlem, New York, with girls seen annually; *b*) Cincinnati Children’s Hospital/University of Cincinnati (Cincinnati), which recruited in the Cincinnati metropolitan area and through the Breast Cancer Registry of Greater Cincinnati, with girls seen semiannually for the first 5 years and then annually thereafter; and *c*) Kaiser Permanente Northern California (KPNC), which recruited KPNC Health Plan members in the San Francisco Bay Area, with girls seen annually. All sites obtained written informed consent from a parent or guardian and were approved by the institutional review board at each site as well as by the Centers for Disease Control and Prevention (CDC). Parents or guardians of the participants identified girls’ race or ethnicity as black, white, Asian, or Hispanic. Standardized anthropometry and pubertal staging were done at each visit (Biro et al. 2010a). Body size characteristics we looked at were height, weight, umbilical waist circumference, and BMI (weight in kilograms divided by squared height in meters). For this analysis we included 940 girls who had anthropometric, dietary, and demographic data with at least three anthropometric measures over the total of 7 years of follow-up (age range, 11–16 years at their last follow-up visit). Height was measured to the nearest 0.1 cm, weight to 0.1 kg, and waist circumference to 0.1 cm. A spot urine sample was collected at baseline for NYC and KPNC and for Cincinnati, at approximately 6 months after baseline and was used to measure the biomarker analytes.

*Laboratory analysis.* Perchlorate, thiocyanate, nitrate, and iodide concentrations in urine were determined by isotope dilution and ion chromatography/tandem mass spectroscopy (IC/MS/MS) as reported previously (Valentin-Blasini et al. 2007). We measured iodide, which is a form of total iodine. Iodide is an inorganic ion used to measure iodine intake, the micronutrient important for thyroid function. Total iodine methods mathematically convert all forms into a single form and report the sum. Therefore, a total iodine measurement includes iodide and a number of organic forms of iodine. Iodide anion accounts for > 90% of total iodine in urine and is the biologically available form that is transported into the thyroid to thyroid hormones (CDC 2008). A total of 940 urine samples were analyzed (312 from NYC, 247 from Cincinnati, and 381 from KPNC). In addition to the internal CDC quality control procedures, we incorporated approximately 10% masked quality control specimens (*n* = 89) from a single urine pool for which the coefficients of variation results were acceptable. Normalization for urine dilution was done by using creatinine-corrected values for perchlorate, thiocyanate, and iodide [micrograms per gram creatinine (gC)] and for nitrate (milligrams per gC). We used this method of correcting for urine dilution to be consistent with others examining NIS inhibitors (Blount 2006). To examine whether results were affected by creatinine adjustment, we analyzed biomarkers uncorrected (micrograms per liter and milligrams per liter) as well as removing extreme creatinine concentrations (< 50 mg/dL and > 300 mg/dL) (Alessio et al. 1985), and we determined that low creatinine values did not produce extreme biomarker concentrations (data not shown). Results based on creatinine-uncorrected values and after excluding samples with extreme values (data not shown) were similar to results from models of creatinine-corrected (reported).

Additional analyses on a subset of 111 girls from the NYC site were performed to assess the intraindividual variation in urinary biomarkers over three time points—at baseline and at 1 and 3 years—using three statistical methods described for a previous analysis of variation over 6 months (Mervish et al. 2012; Teitelbaum et al. 2008). Specifically, we estimated intraclass correlation coefficient (ICC) (Rosner 2000) and Spearman correlation coefficients (SCC), and performed a surrogate category analysis to assess how well tertile ranking by a single biomarker measurement represented average concentrations over 3 years (Hauser et al. 2004; Willett 1998).

*Statistical analyses.* All analyses were conducted using SAS version 9.3 (SAS Institute Inc., Cary, NC). Geometric means and parametric tests of the continuous analyte concentrations were done on natural log–transformed values to achieve a near-normal distribution. Linear mixed-effects models (Proc Mixed in SAS) were used to evaluate associations of one time-point perchlorate, thiocyanate, and nitrate measures with repeated outcome anthropometric measurements (height, waist circumference, weight, and BMI) measured during 3–13 visits. This approach allowed random intercepts and took into account the intrasubject correlation as well as unequal timing of anthropometric measurements. A quadratic term for age was included in the final model because it improved the fit of the model assessed by the Akaike information criterion (AIC). Estimates were compared by testing the difference of the least-square means of the fixed-effects exposure group at each age using the LSMEANS function in Proc Mixed. Statistical significance was defined as *p* < 0.05. We compared the mixed-effects model to a Gompertz model, which has been shown to achieve a reasonably good fit for adolescent human growth (Deming 1957). Results from the Gompertz and mixed-effects model were similar in directionality and effect size (data not shown), which suggests the mixed-effects model adequately described height in our study population.

Covariates considered as potential confounders were selected on a biologic basis or if they were related to both growth and urinary biomarker concentrations [chi-square test, *t*-test, analysis of variance (ANOVA) *p* < 0.05]. Vegetable, dairy, and water consumption are considered the major sources of perchlorate, thiocyanate, and nitrate intake in children (Lau et al. 2013; Murray et al. 2008) and have the potential to be related to growth. In this study population, vegetable, dairy, and water consumption were associated with concentrations of perchlorate and thiocyanate (and nitrate for water) but not with any of the growth outcomes and were therefore were not considered confounders. Additional confounders considered were secondhand smoke exposure (no smoker in the home, smoker in the home, and ≤ or > 35/cigarettes/week) and age at pubertal development (defined as breast Tanner stage 2 and dichotomized at the median of 9.4 years). To investigate differences in growth by site, we included an interaction variable for site by age. Models with and without this interaction term were similar with respect to the AIC; therefore, we present models without the interaction term for ease of interpretation. Because of the complexity of the model, variables that did not alter the coefficients for the association between the exposures categories and the growth outcomes by > 15% or improve the fit of the model by a comparison of the AIC were excluded from the models in a forward selection approach. All final models were adjusted for site and race/ethnicity (indicator terms for black, Hispanic, white, and Asian).

We analyzed the three NIS inhibitors in combination as categorical variables. To investigate possible combined effects of three thyroid disrupting chemicals we created three exposure categories ranked by perchlorate, thiocyanate, and nitrate urinary concentrations We dichotomized at the median perchlorate urinary concentration (5.87 μg/gC), urinary thiocyanate (1,180 μg/gC), and urinary nitrate (676 mg/gC) concentrations. The high-exposure category included girls who had all values above the median for all three NIS inhibitors, the low-exposure category included girls with all values below the median, and the medium-exposure group included the rest of the girls.

## Results

Our sample included 940 girls with baseline urinary biomarkers (median age, 7.3 years) and at least three (range, 3–13) longitudinal anthropometric measures over 7 years of observation. The covariate distributions (urine donation age, race/ethnicity, type of water, vegetable, and dairy consumption) in the full cohort (*n* = 1,239) and in the subsample included in these analyses (*n* = 940) were similar. Table 1 shows urinary exposure biomarker concentrations corrected for creatinine by selected covariates and overall means compared to 6- to 9-year-old girls included in 2007–2008 NHANES (National Health and Nutrition Examination Surveys; http://www.cdc.gov/nchs/nhanes.htm). Concentrations of all NIS biomarkers decreased with age of the girl at urine collection. Urine concentrations of perchlorate, thiocyanate, and nitrate differed significantly among the three sites, with the lowest mean values among NYC participants. Perchlorate and nitrate concentrations decreased with increased BMI percentile, and all NIS inhibitors increased with greater than high school education.

**Table 1:** Geometric means (95% CIs) of creatinine-corrected urine NIS concentrations according to characteristics at baseline among girls in the BCERP cohort (2004–2007, *n* = 940).

Characteristic	*n*	Perchlorate (μg/gC)	Thiocyanate (μg/gC)	Nitrate (mg/gC)	Iodide (μg/gC)
Race/ethnicity
Black	275	4.76 (4.37, 5.18)	1,140 (1,040, 1,260)	604 (567, 645)	179 (164, 194)
Hispanic	285	5.83 (5.36, 6.33)	900 (820, 989)	662 (621, 705)	286 (264, 311)
Asian	53	7.12 (5.87, 8.62)	1,610 (1,290, 2,000)	931 (803, 1E3)	260 (215, 314)
White	327	7.68 (7.11, 8.30)*	1,410 (1,290, 1,540)*	847 (798, 899)*	243 (225, 262)*
Age at urine donation (years)
6–6.99	290	6.44 (5.93, 7.01)	1,150 (1,050, 1,270)	726 (680, 774)	258 (237, 280)
7–7.99	473	6.53 (6.11, 6.97)	1,260 (1,170, 1,360)	753 (716, 793)	237 (222, 253)
≥ 8	177	4.71 (4.23, 5.25)*	964 (853, 1,090)*	610 (562, 663)*	195 (175, 217)*
BMI percentile at urine donation
*< 50th*	644	6.37 (6.02, 6.74)	1,170 (1,100, 1,250)	751 (719, 784)	237 (224, 251)
*50–85th*	142	6.31 (5.59, 7.12)	1,280 (1,110, 1,460)	718 (655, 788)	234 (208, 264)
*> 85th*	154	5.02 (4.47, 5.64)*	1,050 (924, 1,200)	584 (534, 638)*	224 (200, 251)
Caregiver education
≤ High school	272	5.16 (4.73, 5.62)	965 (875, 1,060)	648 (606, 694)	245 (225, 267)
> High school	648	6.66 (6.30, 7.05)*	1,260 (1,180, 1,340)*	746 (714, 779)*	233 (220, 246)
Not reported	20
Site
NYC	312	4.48 (4.14, 4.84)	742 (682, 808)	572 (538, 607)	245 (226, 265)
Cincinnati	247	6.62 (6.07, 7.23)	1,370 (1,240, 1,500)	847 (792, 907)	207 (189, 227)
KPNC	381	7.49 (6.98, 8.04)*	1,520 (1,410, 1,650)*	771 (730, 815)*	245 (228, 264)*
Environmental smoking exposure	
No smokers	695	6.40 (6.06, 6.76)	1,160 (1,090, 1,240)	739 (709, 771)	246 (233, 259)
≥ 1 smoker, 0 cigs	126	5.91 (5.20, 6.72)	1,250 (1,080, 1,440)	716 (649, 791)	215 (189, 243)
≥ 1 smoker, 1–35 cigs/week	48	5.23 (4.25, 6.44)	915 (724, 1,160)	561 (478, 658)	243 (198, 298)
≥ 1 smoker, > 35 cigs/week	65	4.68 (3.91, 5.59)*	1,210 (993, 1,480)	589 (514, 676)*	174 (146, 208)*
Not reported	6
Season of urine donation
Fall	176	5.35 (4.80, 5.96)	1,180 (1,040, 1,330)	668 (614, 726)	213 (191, 238)
Spring	310	6.73 (6.20, 7.31)	1,070 (975, 1,170)	744 (699, 792)	246 (227, 267)
Winter	181	5.67 (5.10, 6.32)	1,030 (917, 1,170)	662 (610, 719)	234 (211, 260)
Summer	273	6.28 (5.75, 6.85)*	1,380 (1,260, 1,530)*	754 (705, 807)*	236 (216, 257)
Primary drinking-water source
50/50 bottle/spring-tap	132	6.24 (5.50, 7.07)	1,260 (1,100, 1,450)	715 (650, 788)	227 (201, 257)
Primarily bottle/spring	263	5.21 (4.77, 5.69)	1,020 (926, 1,130)	626 (585, 670)	222 (203, 243)
Primarily tap	533	6.61 (6.21, 7.04)*	1,210 (1,130, 1,300)*	761 (725, 798)*	244 (229, 260)
Not reported	12
Vegetable consumption tertiles
1 (0.00–0.741 veg/day)	288	5.38 (4.93, 5.86)	1,040 (946, 1,150)	687 (643, 735)	222 (204, 242)
2 (0.741–1.447 veg/day)	289	6.16 (5.65, 6.72)	1,160 (1,050, 1,270)	692 (648, 740)	257 (236, 280)
3 (1.448–5.18 veg/day)	289	6.83 (6.27, 7.45)*	1,330 (1,210, 1,470)*	748 (700, 800)	232 (213, 252)
Not reported	74
Dairy consumption tertiles
1 (0.00–1.576 dairy/day)	288	5.66 (5.18, 6.17)	995 (905, 1,090)	702 (657, 751)	208 (191, 226)
2 (1.577–2.316 dairy/day)	289	6.25 (5.73, 6.82)	1,170 (1,060, 1,280)	717 (671, 766)	244 (224, 265)
3 (2.317–6.85 dairy/day)	289	6.40 (5.87, 6.98)	1,380 (1,260, 1,520)*	707 (662, 756)	261 (240, 284)*
Not reported	74
Age at breast Tanner stage 2 (years)
≤ 9.4	473	5.80 (5.43, 6.20)	1,130 (1,050, 1,220)	707 (671, 744)	226 (211, 241)
> 9.4	433	6.34 (5.91, 6.80)	1,190 (1,100, 1,280)	722 (685, 762)	239 (223, 256)
Not available	34
ALL	940	6.11 (5.83, 6.41)	1,170 (1,110, 1,230)	716 (690, 742)	234 (224, 245)
NHANES^*a*^	284	7.27 (6.56, 8.05)	1,540 (1,370, 1,730)	813 (756, 875)	NA
Abbreviations: cig, cigarettes; NA, not available; veg, vegetables. ^***a***^NHANES 2007–2008, 6- to 9-year-old girls; creatinine-corrected geometric mean (http://www.cdc.gov/nchs/nhanes.html). *ANOVA *p* < 0.05					

It is worth noting that there were no differences in thiocyanate concentrations according to secondhand smoke exposure. There were significant differences in perchlorate and nitrate levels by primary drinking-water source. Perchlorate and thiocyanate significantly differed by vegetable consumption, whereas dairy consumption was only significantly associated with thiocyanate. There were no significant differences in the analyte concentrations by age of pubertal onset. Concentrations among girls in our study were lower than observed among girls ages 6–9 years in 2007–2008 NHANES survey data (http://www.cdc.gov/nchs/nhanes.htm) (Table 1). Geometric means and medians of urinary NIS inhibitors by the three combined NIS exposure categories (low, medium, and high) are shown in Table 2.

**Table 2:** Creatinine-corrected urine iodine and creatinine-corrected urine NIS inhibitor concentrations according to categories of combined NIS exposures (low, medium, and high) in 940 girls (BCERP cohort 2004–2007).

Exposure	*n*	Geo mean	Median	Minimum	Maximum	p95th
Low perchlorate, thiocyanate, and nitrate^*a*^
Perchlorate (μg/gC)	196	3.26	3.59	0.51	5.86	5.5
Thiocyanate (μg/gC)	196	554.61	625.84	75.77	1176.47	1103.4
Nitrate (mg/gC)	196	452.35	500.1	5.92	673.12	644.1
Iodide (μg/gC)	196	176.74	167.7	29.23	2111.11	691.59
Medium perchlorate, thiocyanate, and nitrate
Perchlorate (μg/gC)	555	6.16	5.92	0.25	193.14	18.72
Thiocyanate (μg/gC)	555	1179.71	1201.72	62.34	12756.26	3709.45
Nitrate (mg/gC)	555	734.83	680.25	95.74	129662.5	1895.77
Iodide (μg/gC)	555	235.69	230.26	28.6	4194.92	732.05
High perchlorate, thiocyanate, and nitrate^*b*^
Perchlorate (μg/gC)	189	11.48	9.58	5.87	136.84	35.96
Thiocyanate (μg/gC)	189	2438.13	2343.28	1180.23	24807.69	5675.68
Nitrate (mg/gC)	189	1066.49	955.22	676.47	6647.73	2423.08
Iodide (μg/gC)	189	308.37	294.12	31.71	2636.36	1060.22
Abbreviations: Geo, geometric; p, percentile. ^***a***^Below the median concentration of all three NIS biomarkers. ^***b***^Above the median concentration of all three NIS biomarkers.						

*Intraindividual variability.* The covariate distributions (urine donation age, race/ethnicity, BMI, type of water, vegetable, and dairy consumption) in this subsample (*n* = 111) and in the NYC sample used in the main analyses (*n* = 312) were similar.

However, the girls’ caregivers had lower education compared with the full sample (data not shown). The ICC measure of reproducibility was poor (0.13) for perchlorate, and fair for thiocyanate (0.24), nitrate (0.35), and iodide (0.25) based on creatinine-corrected urine concentrations. However, when average concentrations over all three samples (used as a surrogate indicator of their “true” concentrations over time) were categorized into tertiles according to the distribution of concentrations at each time point, the median value of each tertile increased monotonically from the lowest to the highest tertile (see Supplemental Material, Table S1), thus supporting the use of a single spot urine sample to classify girls into low, medium, and high exposure categories.

**Figure 1:**
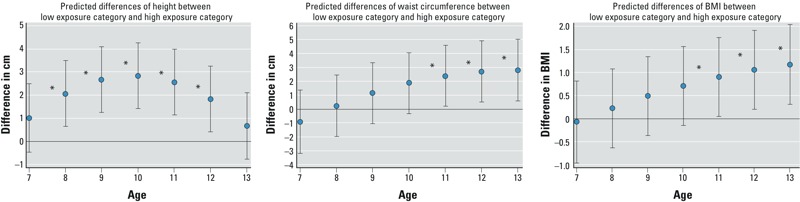
Estimated differences (95% CI) in height, waist circumference, and BMI, by age between the low and high combined NIS biomarker categories: predicted differences using mixed-effects models in 940 girls, BCERP 2004–2007. Low group is below the median concentration of all three NIS biomarkers; high group is above the median concentration of all three NIS biomarkers. Medium group not shown. Final mixed-effects model was adjusted for race/ethnicity and site. *p < 0.05 for the predicted differences between the low- and high-exposure category.

**Figure 2:**
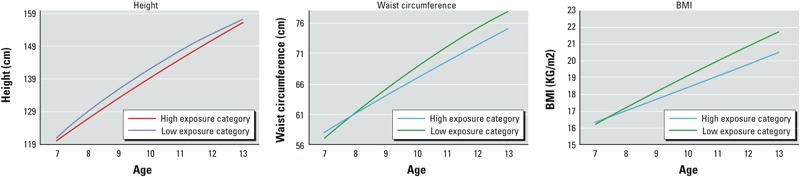
Height, waist circumference, and BMI growth trajectories by age in the low and high combined NIS inhibitor biomarker exposure categories: predicted means using mixed-effects models in 940 girls, BCERP 2004–2007. Low group is below the median concentration of all three NIS biomarkers; high group is above the median concentration of all three NIS biomarkers. Medium group not shown. Growth curves were derived from the LSMEANS function in SAS 9.4 by each exposure category at each age from the final mixed-effects model (adjusted for race/ethnicity and site).

*Growth models*. Height. Girls exposed to combined low concentrations of perchlorate, thiocyanate, and nitrate were consistently taller than the girls with high perchlorate, thiocyanate, and nitrate (Table 3). However, differences in height began to converge by 12 years of age, and heights were similar between the high- and low-exposure groups by 13 years of age (Figures 1 and 2).

**Table 3:** Height, waist circumference, and BMI by age in low, medium, and high combined NIS inhibitor biomarker exposure categories*^a^*: predicted means and differences (95% CIs) based on mixed effects models*^b^* in 940 girls, BCERP 2004–2007.

Age (years)^*c*^	Low-exposure category	Medium-exposure category	High-exposure category	Difference between low and high (95% CI)	*p*-Value^*d*^
Height (cm)
7	121.2 (120.1, 122.2)	120.6 (120.0, 121.3)	120.1 (119.1, 121.2)	1.0 (–0.4, 2.5)	0.172
8	129.0 (127.9, 130.0)	127.8 (127.2, 128.5)	126.9 (125.9, 127.9)	2.1 (0.6, 3.5)	0.004
9	136.0 (135.0, 137.1)	134.5 (133.9, 135.2)	133.4 (132.3, 134.4)	2.7 (1.3, 4.1)	< 0.001
10	142.4 (141.3, 143.4)	140.8 (140.1, 141.4)	139.5 (138.5, 140.6)	2.8 (1.4, 4.3)	< 0.001
11	148.0 (146.9, 149.0)	146.5 (145.9, 147.2)	145.4 (144.4, 146.4)	2.6 (1.1, 4)	< 0.001
12	152.8 (151.8, 153.9)	151.8 (151.1, 152.5)	151.0 (150.0, 152.0)	1.8 (0.4, 3.3)	0.011
13	157.0 (155.9, 158.0)	156.6 (155.9, 157.3)	156.3 (155.2, 157.3)	0.7 (–0.8, 2.1)	0.356
Waist circumference (cm)
7	57.1 (55.5, 58.8)	58.7 (57.7, 59.8)	58.0 (56.4, 59.7)	–0.9 (–3.2, 1.4)	0.444
8	61.3 (59.7, 62.9)	62.2 (61.2, 63.2)	61.1 (59.5, 62.7)	0.2 (–2, 2.4)	0.830
9	65.2 (63.6, 66.8)	65.6 (64.6, 66.6)	64.1 (62.5, 65.7)	1.2 (–1, 3.3)	0.295
10	68.8 (67.2, 70.4)	68.8 (67.8, 69.8)	67.0 (65.4, 68.5)	1.9 (–0.3, 4.1)	0.091
11	72.1 (70.5, 73.8)	71.9 (70.9, 72.9)	69.7 (68.2, 71.3)	2.4 (0.2, 4.6)	0.032
12	75.2 (73.5, 76.8)	74.9 (73.9, 75.9)	72.4 (70.9, 74.0)	2.7 (0.5, 4.9)	0.016
13	77.9 (76.2, 79.5)	77.7 (76.7, 78.7)	75.0 (73.4, 76.6)	2.8 (0.6, 5)	0.013
BMI (kg/m^2^)
7	16.3 (15.6, 16.9)	16.7 (16.3, 17.1)	16.4 (15.7, 17.0)	–0.1 (–1, 0.8)	0.871
8	17.3 (16.6, 17.9)	17.5 (17.1, 17.9)	17.0 (16.4, 17.6)	0.2 (–0.6, 1.1)	0.604
9	18.2 (17.6, 18.8)	18.3 (17.9, 18.7)	17.7 (17.1, 18.3)	0.5 (–0.4, 1.3)	0.259
10	19.1 (18.5, 19.7)	19.1 (18.7, 19.5)	18.4 (17.8, 19.0)	0.7 (–0.1, 1.6)	0.100
11	20.0 (19.4, 20.6)	20.0 (19.6, 20.4)	19.1 (18.5, 19.7)	0.9 (0.1, 1.8)	0.038
12	20.9 (20.2, 21.5)	20.8 (20.4, 21.2)	19.8 (19.2, 20.4)	1.1 (0.2, 1.9)	0.015
13	21.7 (21.1, 22.3)	21.7 (21.3, 22.1)	20.5 (19.9, 21.2)	1.2 (0.3, 2)	0.008
^***a***^Low group is below the median concentration of all three NIS biomarkers; high group is above the median concentration of all three NIS biomarkers; medium group includes all the others. ^***b***^Predicted means and differences were computed from the final model (adjusted for race/ethnicity and site) using the LSMEANS function in Proc Mixed SAS version 9.4. ^***c***^No estimate was provided for age 6 years due to small sample of girls who were exactly 6 years old at enrollment, truncated at age 13. ^***d***^Significance of the difference between the low- and high-exposure category.					

Waist circumference and BMI. Waist circumference and BMI models showed similar patterns, such that after age 7 years, girls with low exposures had larger values than girls in the medium- and high-exposure groups, respectively, though differences between the low- and high-exposure groups were not statistically significant until 11 years of age (Table 3). In addition, differences between the low- and high-exposure groups increased with age (Figures 1 and 2).

## Discussion

To our knowledge, this is the first study to examine either individual or combined effects of NIS inhibitors on body size and growth during childhood. High exposure to all three NIS inhibitors combined (perchlorate, thiocyanate, and nitrate urinary concentrations) showed significant differences in growth outcomes at various ages compared with low exposure to all three NIS inhibitors. Our results are consistent with these chemicals as thyroid antagonists, in that higher exposure to the three NIS inhibitors was associated with smaller mean body size measurements at each age and slower growth in girls. Beginning at 11 years of age, the predicted difference was significant for mean waist circumference and BMI between the low- and high-exposure groups. Known sources of exposure and factors associated with growth in children (vegetable, dairy, and water consumption) were examined as potential confounders but did not alter associations (data not shown). Chronically impaired iodide uptake is associated with alterations in thyroid hormone levels (Blount et al. 2006; Steinmaus et al. 2007, 2013). However in our population, iodide levels were generally adequate so that observed associations between exposures and body size measures may be through another mechanism. It is also possible that the various chemicals compete for iodide, but do not necessarily deplete iodide. Greer et al. (2002) suggested that intermittent periods of low iodide intake may not affect thyroid hormone production.

Chemicals may affect different growth indices during distinct periods of a child’s life. Our study examined the prepubertal and pubertal time periods. Early-maturing girls have significantly higher BMI and percent body fat than on-time and later-maturing girls (Biro et al. 2010b). When we included the mean age at pubertal development in our models, the association remained the same (data not shown). The trajectories of waist circumference (WC) and BMI are consistent with growth during puberty; as heavier girls grow older, there is also a greater increase in adiposity compared with lighter girls (Biro and Wein 2010). WC and BMI have been used as a means of identifying children at risk for hypertension, type 2 diabetes, and cardiovascular disease in both childhood and later as adults (Czernichow et al. 2011b; Freedman et al. 2007). More recently, WC, a measure of visceral fat deposits and used for defining abdominal obesity, is shown to be better than BMI for predicting those conditions in children, including metabolic syndrome and all-cause mortality (Czernichow et al. 2011a; Xi et al. 2014). In this population, WC, BMI, and weight are all highly correlated with each other (*r* > 0.90).

Studies show elevated thyroid hormone levels [TSH, T_4_, and triiodothyronine (T_3_)] in obese children and adults compared with normal weight persons (Chomard et al. 1985; Gussekloo et al. 2004; Knudsen et al. 2005; Matzen et al. 1989; Reinehr and Andler 2002; Shalitin et al. 2009). An association between thyroid function and BMI may be attributable to alterations in energy expenditure or leptin produced by adipocyte tissue, although the mechanism is unclear (Reinehr 2010). Controversy exists as to whether the changes in TSH or other thyroid hormones are causes or consequences of weight status.

Perchlorate associations with thyroid hormone production have been studied in infants and children with mixed results (Brechner et al. 2000; Crump et al. 2000; Lamm 2003; Li et al. 2001; Téllez Téllez et al. 2005). Thyroid deficiency is of particular concern during development because these hormones regulate brain development (Haddow et al. 1999). Several studies of NIS inhibitors on thyroid hormones have estimated exposure based on perchlorate levels measured in water supplies. Most have not reported associations with thyroid hormone concentrations on either the pregnant mother or neonate (Amitai et al. 2007; Crump et al. 2000; Li et al. 2001; Téllez Téllez et al. 2005); however, one study reported elevated TSH levels in newborns whose drinking-water supply was perchlorate-contaminated compared with those supplied with drinking water that had no perchlorate contamination (Brechner et al. 2000). Several experimental and observational studies using urinary perchlorate biomarkers in adults have reported that perchlorate was not associated with T_4_ or TSH levels at exposure levels orders of magnitude higher than the median levels found in our study (Braverman et al. 2005, 2006; Lamm et al. 1999; Lamm 2003). One study found perchlorate negatively associated with T_4_ only in women with iodine < 100 μg/L (Blount et al. 2006), suggesting that iodine levels must be sufficiently low for environmental levels of perchlorate and thiocyanate to overcome compensatory mechanisms that maintain thyroid hormone (Steinmaus et al. 2007), at least in adults.

Previous research on perchlorate and possible thyroid-related health effects has paid little attention to the other common environmental NIS inhibitors, thiocyanate and nitrate. The focus on perchlorate arises in part because its relative potency as an NIS inhibitor is 10–200 times that of thiocyanate and nitrate on a molar basis (Greer et al. 1966; Tonacchera et al. 2004). However, based on average daily intake of percholorate equivalents of nitrates and thiocyanates, a person’s exposure to both thiocyanate and nitrate from drinking water and food account for a larger proportion of iodine uptake inhibition than does perchlorate exposure (De Groef et al. 2006). Moreover, *in vitro* studies of NIS indicate that perchlorate, nitrate, and thiocyanate act additively to inhibit iodide uptake (Tonacchera et al. 2004). Therefore, it seems important to study the combination of chemicals.

Nitrate is a natural common chemical contaminant from both food and drinking-water intake, and vegetables account for between 30% and 80% of the total nitrate intake (Du et al. 2007). High levels of nitrate (> 200 mg/L) in drinking water have been associated with goiter incidence (Gatseva and Argirova 2008), increased thyroid volume (van Maanen et al. 1994), and subclinical thyroid disorders (Tajtáková et al. 2006). A study with lower levels than the above studies and ours—mean, 53 mg/L—found no association with thyroid volume (Below et al. 2008). Although nitrate itself has no toxicity, its metabolites, nitrite and N-nitroso compounds (NOCs), can be toxic. On the other hand, NOCs, which can be produced in the stomach, have multiple physiological roles, some of which are positive (Du et al. 2007). Thiocyanate has a low toxicity and can be found as both a metabolite of cyanide, which enters the body mainly from tobacco smoke, and free thiocyanate, as found in vegetables. The latter form has antibacterial properties (Youso et al. 2012). The fact that nitrates and thiocyanate are capable of both deleterious and beneficial effects calls for a better understanding of factors associated with their metabolism. Moreover, because vegetable, dairy, and water consumption are major sources of NIS inhibitors (De Groef et al. 2006; Lau et al. 2013; Murray et al. 2008), the risks and benefits of exposure to NIS inhibitors need to be carefully considered.

There is increasing evidence linking environmental toxicants to thyroid dysfunction. Studies have reported associations between thyroid hormone levels and exposure to polychlorinated biphenyls (PCBs) (Schell et al. 2008), phthalates (Meeker et al. 2007), perchlorate (NRC 2005), and bisphenol A (BPA) (Meeker et al. 2010). Mechanisms underlying thyroid interference by these chemicals are diverse, and include increased inhibition of TH synthesis or increased metabolism of THs via induction of deiodinases (Zoeller 2007). These same chemicals have also been associated with body size, including phthalates (Hatch et al. 2008; Stahlhut et al. 2007) and BPA (Rubin and Soto 2009), potentially through the thyroid mechanism (Newbold et al. 2008).

A limitation of our study is using one spot urine sample, because these chemicals have short half-lives in the body (< 1 day, except thiocyanate, which is about 6 days) and may not represent one’s long-term exposure. This study and an earlier investigation (Mervish et al. 2012) examined repeatability of measures over 3 years and 6 months, respectively. Here, we showed poor to fair agreement (ICCs between 0.13–0.35) of NIS concentrations in samples over 3 years, comparable with results from the 6-month interval (Mervish et al. 2012). The use of a single spot urine sample would most likely lead to nondifferential misclassification. We also had no information on maternal BMI, birth weight, and other maternal indices associated with their offspring’s childhood growth (Laitinen et al. 2001; Terry et al. 2007). An additional limitation is our inability to directly investigate the proposed pathway involving the thyroid because we do not have thyroid hormone measures.

Strengths of this prospective study include longitudinal standardized measures of several parameters of growth. We have at least 3 and as many as 13 anthropometric measurements to examine longitudinal growth. We used mixed-effects growth curve modeling, which allows examination of timing of differences in growth. We were able to investigate the combined effects of three thyroid-disrupting agents—animal and laboratory studies have shown that they act in combination to affect thyroid hormone production (De Groef et al. 2006).

Although growth was associated with exposure to NIS inhibitors in our study, growth is a result of a complex interaction between genetic and environmental factors and not attributable solely to any one factor. Given the limitations of having one spot urine sample, no thyroid measures, and low exposures, these findings need to be further investigated. Nonetheless, changes in growth due to these NIS inhibitors are biologically plausible because normal somatic growth requires the thyroid hormone axis be intact, and alterations in thyroid hormones can potentially affect body size changes (Zimmermann 2007). Studying potential influences of childhood growth is important because childhood growth is predictive of adult disease and mortality (Freedman et al. 2007). However, it is unclear whether the differences in growth associated with exposure in our study population have implications for future growth and health.

## Supplemental Material

(123 KB) PDF
