# Supplementary material for: Thyroid Antagonists (Perchlorate, Thiocyanate, and Nitrate) and Childhood Growth in a Longitudinal Study of U.S. Girls
Source: Environ Health Perspect. 2015 Jul 7;124(4):542–9. doi: 10.1289/ehp.1409309 (PMC4829993; doi:10.1289/ehp.1409309)
Supplement: (123 KB) PDF [file ehp.1409309.s001.acco.pdf]

Environ Health Perspect
DOI: 10.1289/ehp.1409309

Note to Readers: *EHP* strives to ensure that all journal content is accessible to all readers. However, some figures and Supplemental Material published in *EHP* articles may not conform to 508 standards due to the complexity of the information being presented. If you need assistance accessing journal content, please contact ehp508@niehs.nih.gov. Our staff will work with you to assess and meet your accessibility needs within 3 working days.

Supplemental Material

Thyroid Antagonists (Perchlorate, Thiocyanate, and Nitrate) and Childhood Growth in a Longitudinal Study of U.S. Girls

Nancy A. Mervish, Ashley Pajak, Susan L. Teitelbaum, Susan M. Pinney, Gayle C. Windham, Lawrence H. Kushi, Frank M. Biro, Liza Valentin-Blasini, Benjamin C. Blount, and Mary S. Wolff, [1] for the Breast Cancer and Environment Research Project (BCERP)

Table of Contents

Table S1. Three-year geometric mean of creatinine corected perchlorate, nitrate, thiocyanate and iodide urine concentrations within surrogate sample determined exposure tertiles[a].

Table S1. Three-year geometric mean of creatinine corected perchlorate, nitrate, thiocyanate and iodide urine concentrations within surrogate sample determined exposure tertiles[a].

	Geometric mean (ug/g Creatinine)		
**Surrogate Sample**	**Low**	**Medium**	**High**
**Perchlorate**			
Baseline	3.33	4.11	5.87
Year 1	3.18	4.29	5.83
Year 3	3.07	4.58	5.67
**Nitrate (mg/g creatinine)**			
Baseline	422	576	768
Year 1	422	593	746
Year 3	417	564	794
**Thiocyanate**			
Baseline	463	713	1067
Year 1	460	680	1100
Year 3	471	698	1048
**Iodide**			
Baseline	127	195	324
Year 1	132	200	304
Year 3	129	210	295

[a]Analyses were conducted on data from 111 girls with three samples collected at baseline, 1 and 3 years after.

The concentration distribution of the 111 samples collected at each time point were used to create tertile cutpoints for assignment of each child into low, medium, or high exposure level. Mean concentrations (low, medium and high) were calculated using the three-year means of the individual children assigned to each exposure group (N≅37). All calculations were performed on natural-log transformed data and were back transformed to obtain geometric means.
